# Supplementary material for: DIP1 modulates stem cell homeostasis in Drosophila through regulation of sisR-1
Source: Nat Commun. 2017 Oct 2;8:759. doi: 10.1038/s41467-017-00684-4 (PMC5624886; doi:10.1038/s41467-017-00684-4)
Supplement: Supplementary file 1 — Supplementary Information [file 41467_2017_684_MOESM1_ESM.pdf]

Description of Supplementary Files

File name: Supplementary Information
Description: Supplementary figures.

File name: Peer review file

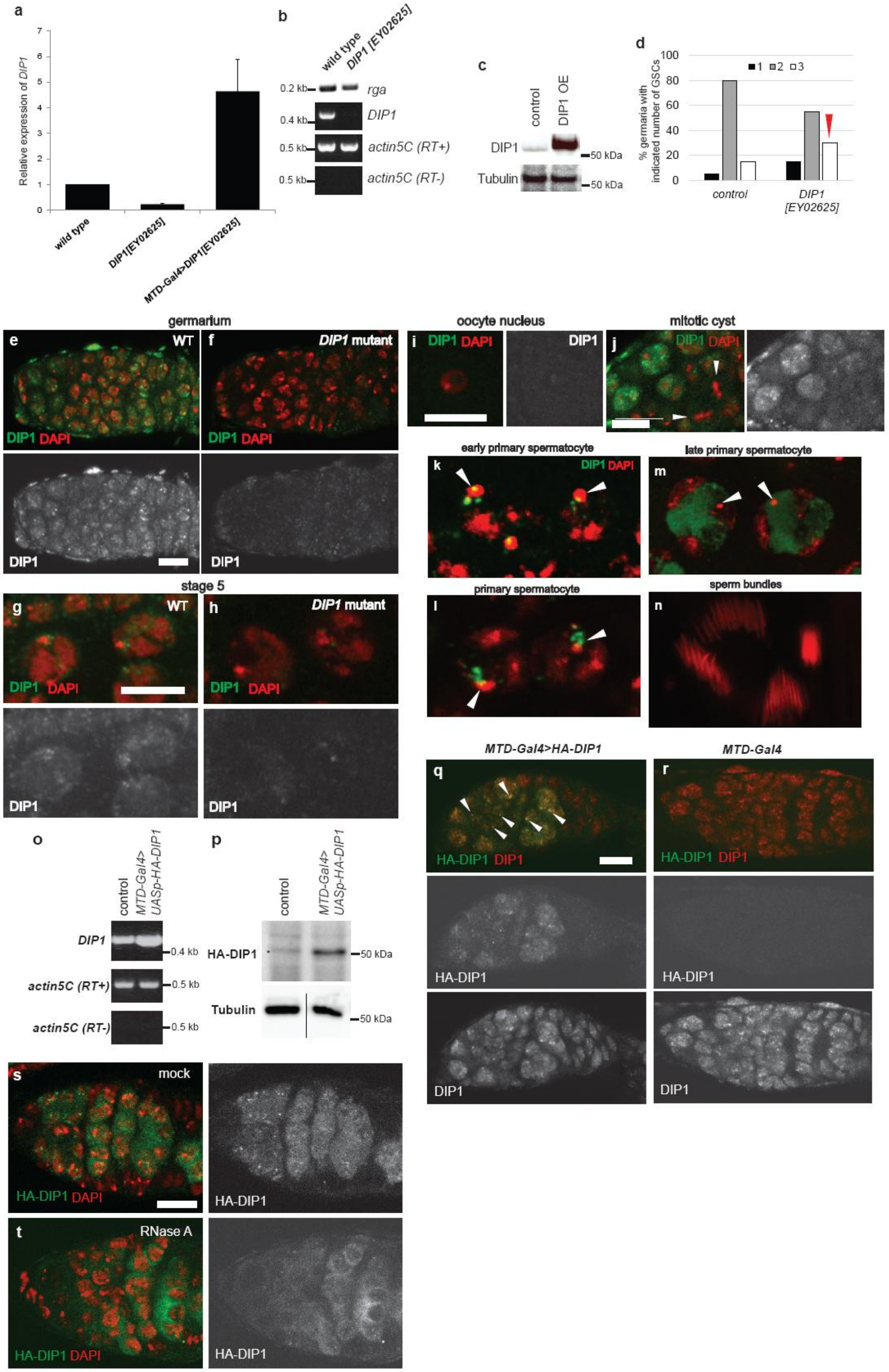
a
Relative expression of DIP1
0
1
2
3
4
5
6
7
wild type
DIP1[EY02625]
MTD-Gal4>DIP1[EY02625]
b
wild type
DIP1 [EY02625]
0.2 kb
rga
0.4 kb
DIP1
0.5 kb
actin5C (RT+)
0.5 kb
actin5C (RT-)
c
control
DIP1 OE
DIP1
Tubulin
50 kDa
50 kDa
d
% germaria with indicated number of GSCs
100
80
60
40
20
0
1
2
3
control
DIP1 [EY02625]
germarium
e
WT
DIP1 DAPI
DIP1
f
DIP1 mutant
DIP1 DAPI
DIP1
stage 5
g
WT
DIP1 DAPI
DIP1
h
DIP1 mutant
DIP1 DAPI
DIP1
oocyte nucleus
i
DIP1 DAPI
DIP1
mitotic cyst
j
DIP1 DAPI
early primary spermatocyte
k
DIP1 DAPI
late primary spermatocyte
m
primary spermatocyte
l
sperm bundles
n
o
control
MTD-Gal4> UASp-HA-DIP1
DIP1
0.4 kb
actin5C (RT+)
0.5 kb
actin5C (RT-)
0.5 kb
p
control
MTD-Gal4> UASp-HA-DIP1
HA-DIP1
50 kDa
Tubulin
50 kDa
MTD-Gal4>HA-DIP1
q
HA-DIP1 DIP1
HA-DIP1
DIP1
MTD-Gal4
r
HA-DIP1 DIP1
HA-DIP1
DIP1
s
mock
HA-DIP1 DAPI
HA-DIP1
t
RNase A
HA-DIP1 DAPI
HA-DIP1

Supplementary Figure 1. Characterization of DIP1. (a) RT-qPCR showing the relative expression of *DIP1* mRNA in the indicated genotypes. (b) RT-PCR showing the expression of *rga* in the ovaries of wild type versus *DIP1*^{EY02625} mutant ovaries. (c) Western blot showing specificity of DIP1 antibody. (d) Chart showing the percentages of germaria with the indicated number of GSCs of the indicated genotypes. Red arrowhead indicates the increase in germaria with 3 GSCs. (e-h) DIP1 localizes as foci in the nuclei of the germline cells in the ovaries. (e, f) Staining of DIP1 in the germaria of (e) wildtype and (f) *DIP1* mutants. (g, h) Staining of DIP1 in the stage 5 egg chambers of (g) wildtype and (h) *DIP1* mutants. (i, j) Staining of DIP1 in the (i) oocyte nucleus and (j) germline cells undergoing mitosis (arrowheads indicate 2 cells at metaphase). (k-n) DIP1 localizes as foci in the nuclei of the germline cells in the testes. Staining of DIP1 in the (k) early primary spermatocytes, (l) primary spermatocytes, (m) late primary spermatocytes, and (n) sperm bundles. Arrowheads point to DAPI-dense regions of fourth chromosomes. Scale bar: 10 µm. (o-p) Characterization of HA-DIP1 overexpression flies. (o) RT-PCR showing the expression of DIP1 in controls (*MTD-Gal4*) and HA-DIP1 overexpression (*MTD-Gal4>UASp-HA-DIP1*) flies. *Actin5C* was used as a loading control. (p) Western blot showing the expression of HA-DIP1 protein in controls (*MTD-Gal4*) and HA-DIP1 overexpression (*MTD-Gal4>UASp-HA-DIP1*) flies. Alpha-Tubulin was used as a loading control. (q, r) Staining of HA and DIP1 in (q) *MTD-Gal4>HA-DIP1* and (r) *MTD-Gal4* control ovaries. Arrowheads point to co-localized foci. (s, t) Staining of DIP1 in (s) mock treated and (t) RNase A treated ovaries. Scale bar: 10 µm.

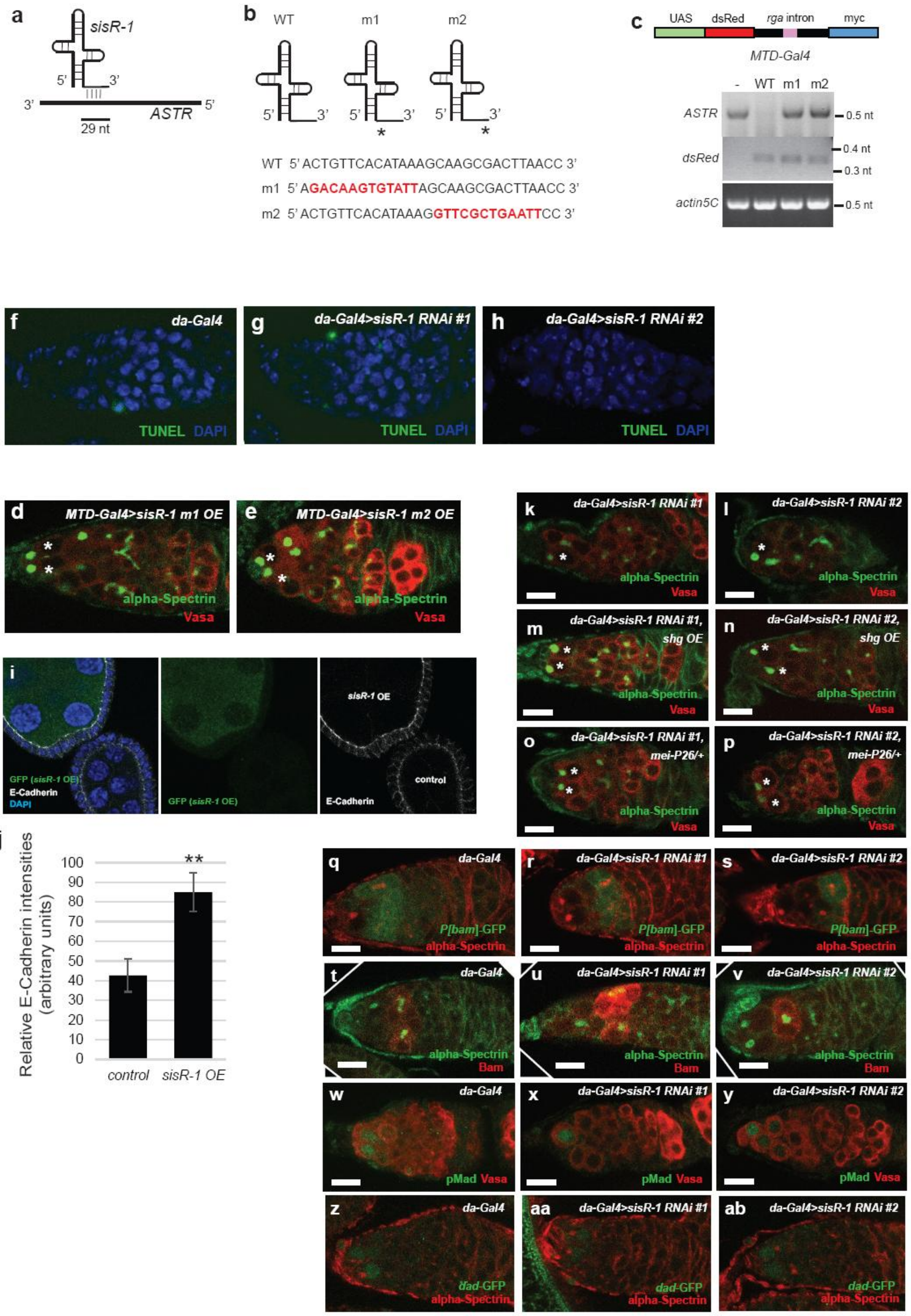
a
sisR-1
5'
3'
3'
5'
ASTR
29 nt
b
WT
m1
m2
WT 5' ACTGTTCACATAAAGCAAGCGACTTAACC 3'
m1 5' AGACAAGTGTATTAGCAAGCGACTTAACC 3'
m2 5' ACTGTTCACATAAAGGTTCGCTGAATTCC 3'
c
UAS
dsRed
rga intron
myc
MTD-Gal4
-
WT
m1
m2
ASTR
dsRed
actin5C
0.5 nt
0.4 nt
0.3 nt
0.5 nt
f
da-Gal4
TUNEL DAPI
g
da-Gal4>sisR-1 RNAi #1
TUNEL DAPI
h
da-Gal4>sisR-1 RNAi #2
TUNEL DAPI
d
MTD-Gal4>sisR-1 m1 OE
alpha-Spectrin
Vasa
e
MTD-Gal4>sisR-1 m2 OE
alpha-Spectrin
Vasa
k
da-Gal4>sisR-1 RNAi #1
alpha-Spectrin
Vasa
l
da-Gal4>sisR-1 RNAi #2
alpha-Spectrin
Vasa
m
da-Gal4>sisR-1 RNAi #1,
shg OE
alpha-Spectrin
Vasa
n
da-Gal4>sisR-1 RNAi #2,
shg OE
alpha-Spectrin
Vasa
o
da-Gal4>sisR-1 RNAi #1,
mei-P26/+
alpha-Spectrin
Vasa
p
da-Gal4>sisR-1 RNAi #2,
mei-P26/+
alpha-Spectrin
Vasa
i
GFP (sisR-1 OE)
E-Cadherin
DAPI
GFP (sisR-1 OE)
sisR-1 OE
control
E-Cadherin
j
Relative E-Cadherin intensities
(arbitrary units)
100
90
80
70
60
50
40
30
20
10
0
**
control
sisR-1 OE
q
da-Gal4
P[bam]-GFP
alpha-Spectrin
r
da-Gal4>sisR-1 RNAi #1
P[bam]-GFP
alpha-Spectrin
s
da-Gal4>sisR-1 RNAi #2
P[bam]-GFP
alpha-Spectrin
t
da-Gal4
alpha-Spectrin
Bam
u
da-Gal4>sisR-1 RNAi #1
alpha-Spectrin
Bam
v
da-Gal4>sisR-1 RNAi #2
alpha-Spectrin
Bam
w
da-Gal4
pMad Vasa
x
da-Gal4>sisR-1 RNAi #1
pMad Vasa
y
da-Gal4>sisR-1 RNAi #2
pMad Vasa
z
da-Gal4
dad-GFP
alpha-Spectrin
aa
da-Gal4>sisR-1 RNAi #1
dad-GFP
alpha-Spectrin
ab
da-Gal4>sisR-1 RNAi #2
dad-GFP
alpha-Spectrin

Supplementary Figure 2. Characterization of *sisR-1*. (a-c) Overexpression of *sisR-1* with mutated 3' ends. (a) Hypothetical model of *sisR-1* mediated silencing of *ASTR* via complementary base-pairing of its 3' end with *ASTR*. (b) Diagram showing the WT and different mutant forms of *sisR-1* generated. Mutated sequences in m1 and m2 were in red. (c) RT-PCR showing the expressions of *ASTR* and *dsRed* transgenes in the ovaries of the indicated genotypes. *Actin5C* was used as a loading control. Mutations were made in the pink region in the *rga* intron in the *UAS-dsRed-intron-myc* construct. (d, e) Confocal images of germaria of the indicated genotypes stained for alpha-Spectrin (green) and Vasa (red). Scale bar: 10µM. Asterisks (*) mark the GSCs. (f-h) Apoptosis in *sisR-1* RNAi ovaries. Confocal images of germaria of the indicated genotypes stained for apoptotic cells (green) and DAPI (Blue). (i) Immunostaining of egg chambers showing expression of GFP (green, *sisR-1* overexpression clone), E-Cadherin (white) and DAPI (blue). (j) Chart showing the quantification of E-Cadherin intensities. **$p<0.01$. N=3. (k-p) Genetic interactions between *sisR-1* and *shg* and *mei-P26*. Confocal images of germaria of the indicated genotypes stained for alpha-Spectrin (green) and Vasa (red). Scale bar: 10µM. Asterisks (*) mark the GSCs. (q-ab) Effects of *sisR-1* RNAi on *dpp* signalling and *bam* expression. (q-s) Confocal images of germaria of the indicated genotypes stained for *P[bam]*-GFP (green) and alpha-Specrtin (red). (t-v) Confocal images of germaria of the indicated genotypes stained for alpha-Spectrin (green) and Bam (red). (w-y) Confocal images of germaria of the indicated genotypes stained for pMad (green) and Vasa (red). (z-ab) Confocal images of germaria of the indicated genotypes stained for *dad*-GFP (green) and alpha-Spectrin (red). Scale bar: 10µM.

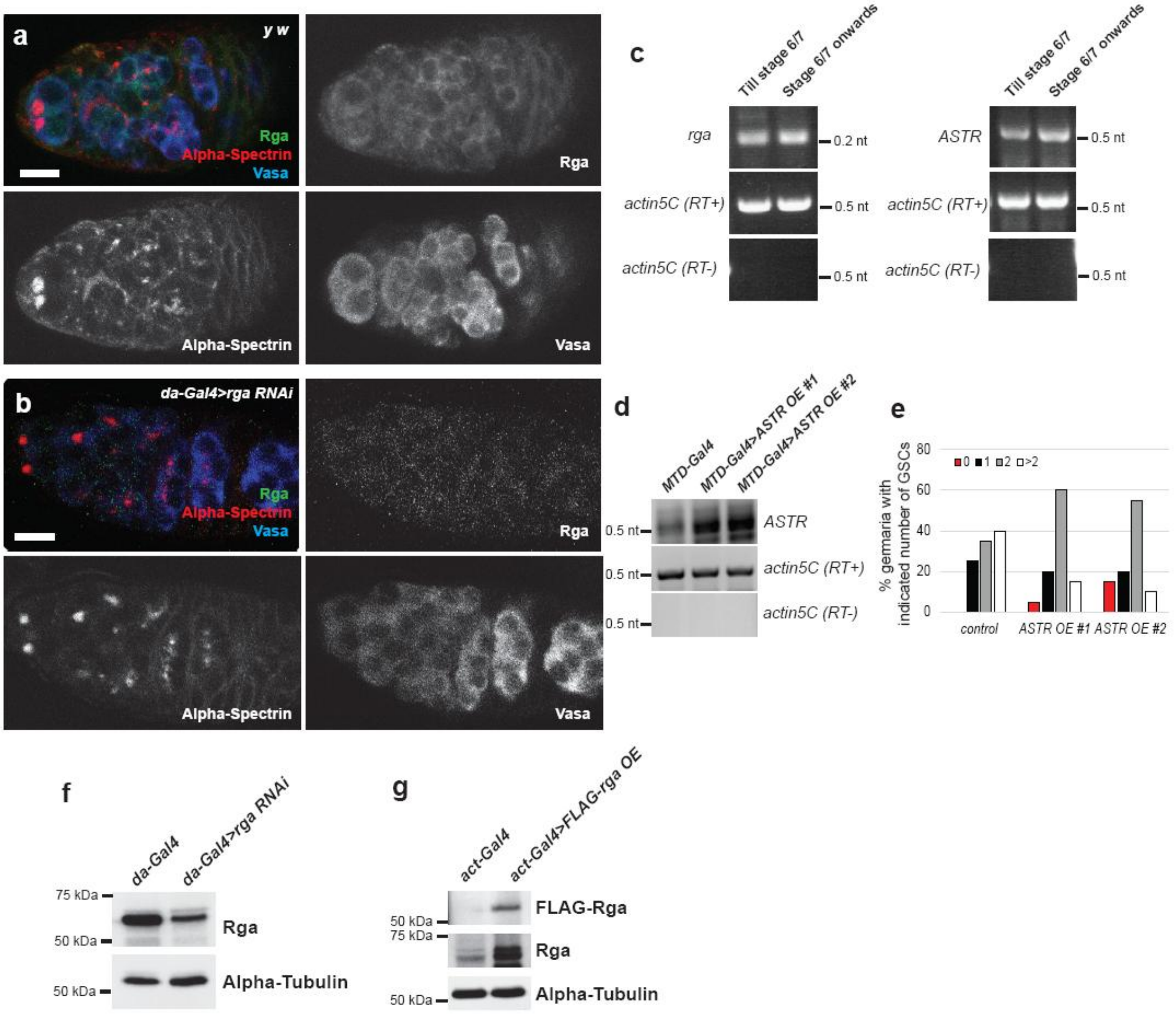

Supplementary Figure 3. Characterization of *ASTR* and *rga*. (a-c) Expression of *ASTR* and *rga*. (a, b) Confocal images of germaria of the indicated genotypes stained for Rga (green), alpha-Spectrin (red) and Vasa (blue). (c) RT-PCR showing expression of *rga* and *ASTR* in both the early (till stage 6/7) and late (stage 6/7 onwards) stage ovaries. (d, e) Overexpression of *ASTR*. (d) RT-PCR showing the expression of *ASTR* in the ovaries of the indicated genotypes. *Actin5C* was used as a loading control. (e) Chart showing the percentages of germaria with the indicated number of GSCs of the indicated genotypes. (f) Western blot showing the expression of Rga protein in the ovaries of the indicated genotypes. Alpha-Tubulin was used as a loading control. (g) Western blot showing the expression of FLAG-Rga and Rga protein in the ovaries of the indicated genotypes.

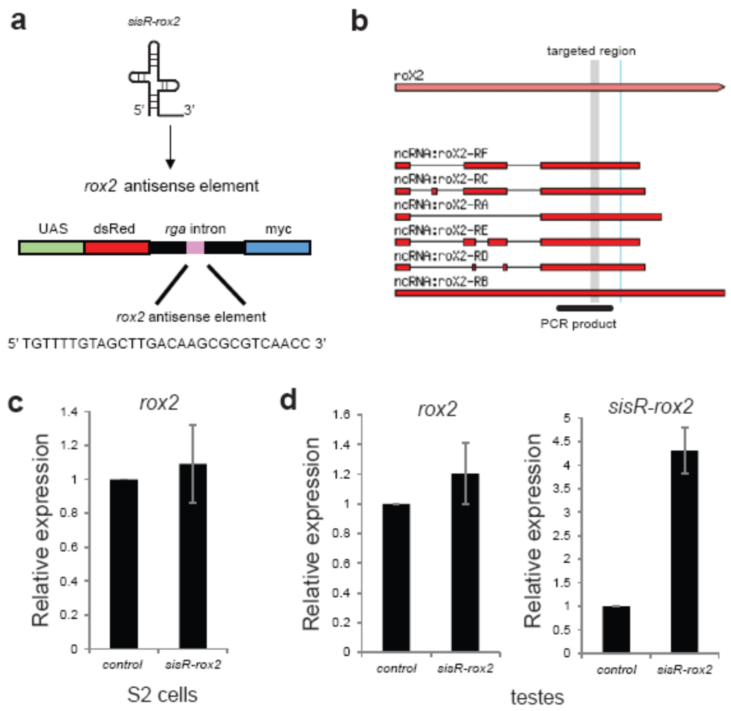

Supplementary Figure 4. The 3' tail of *sisR-1* is not sufficient for silencing. (a) Drawing showing the replacement of 3' tail with an antisense element against *rox2*. (b) Drawing showing the region of *rox2* RNA targeted by the antisense element in (a). Black bar indicates region amplified by PCR in (c) and (d). (c) RT-qPCR showing the relative expression of *rox2* in non-transfected control versus *sisR-rox2* transfected S2 cells. (d) RT-qPCR showing the relative expression of *rox2* and *sisR-rox2* in control versus testes ectopically expressing *sisR-rox2*.

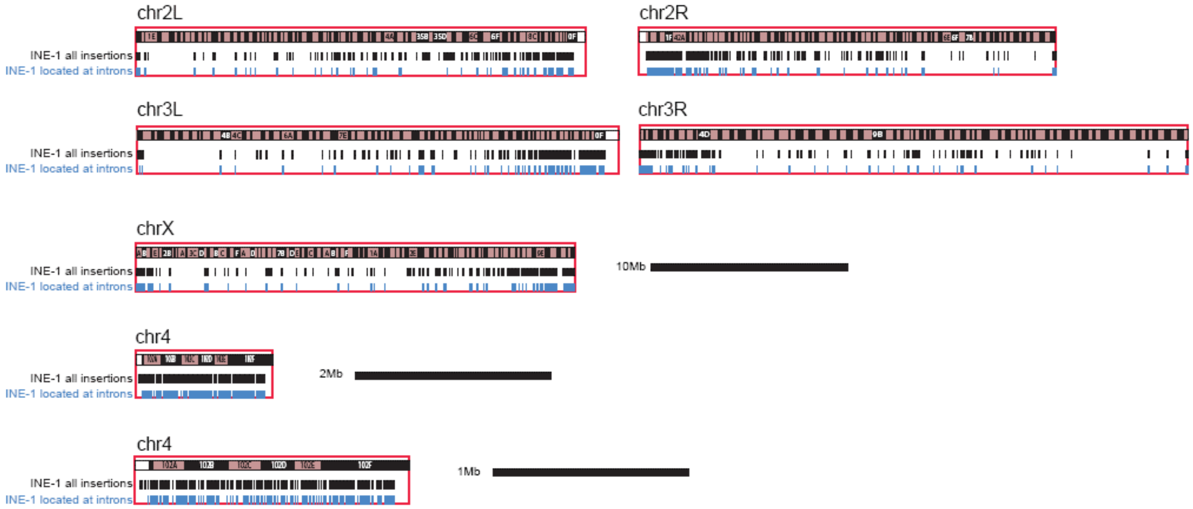

Supplementary Figure 5. Distribution of INE-1 insertions (all and only in introns) over the chromosomes in *Drosophila melanogaster*.

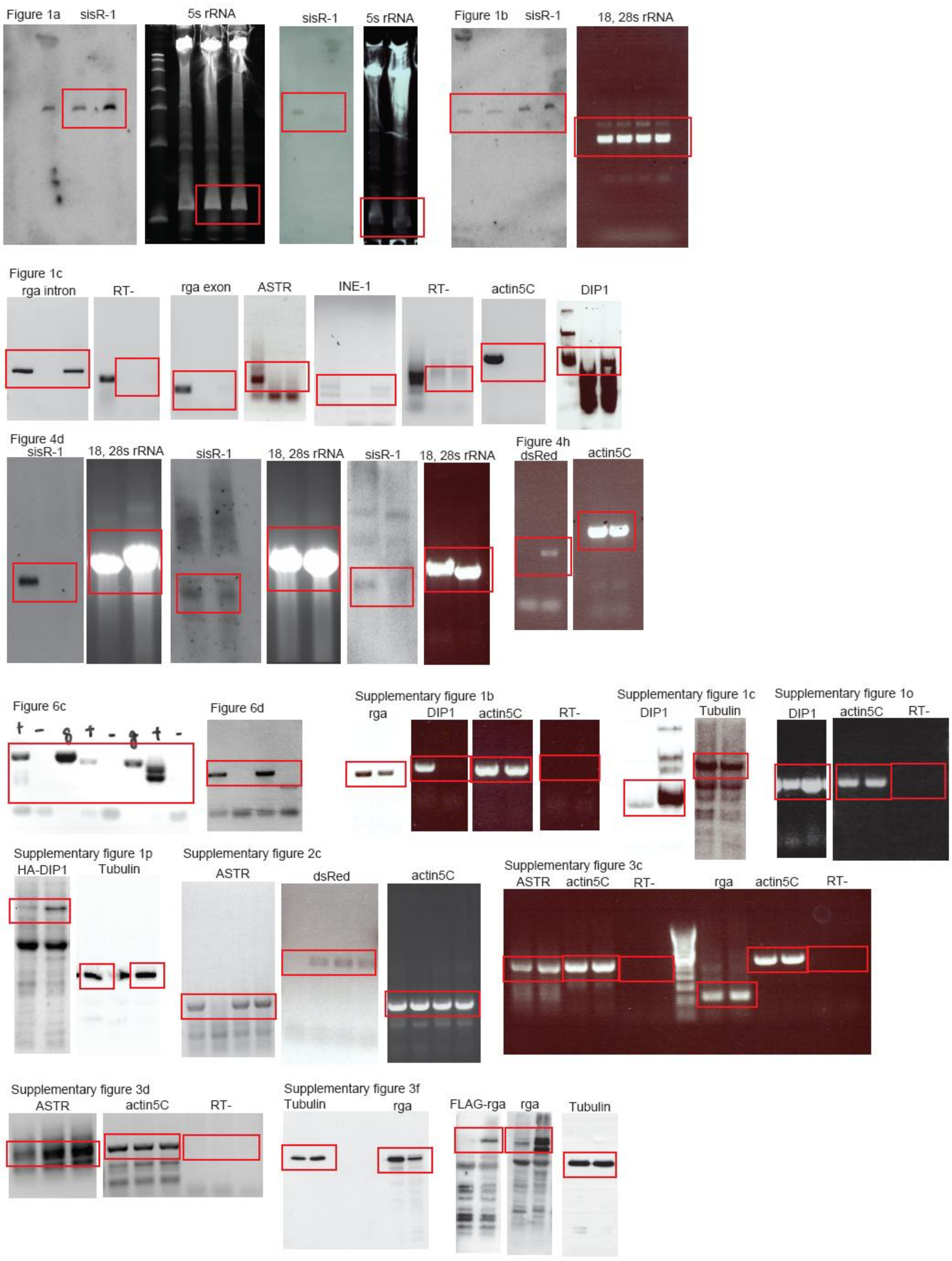

Supplementary Figure 6. Original blots and gels used in the main and supplementary figures.
